# Cancer carrier screening in the general population using whole‐genome sequencing

**DOI:** 10.1002/cam4.5034

**Published:** 2022-07-21

**Authors:** Ya‐Sian Chang, Dy‐San Chao, Chin‐Chun Chung, Yu‐Pao Chou, Chieh‐Min Chang, Chia‐Li Lin, Hou‐Wei Chu, Hon‐Da Chen, Ting‐Yuan Liu, Yu‐Hsuan Juan, Shun‐Jen Chang, Jan‐Gowth Chang

**Affiliations:** ^1^ Center for Precision Medicine China Medical University Hospital Taichung Taiwan; ^2^ Epigenome Research Center China Medical University Hospital Taichung Taiwan; ^3^ Department of Laboratory Medicine China Medical University Hospital Taichung Taiwan; ^4^ School of Medicine China Medical University Taichung Taiwan; ^5^ Institute of Biomedical Sciences| Academia Sinica Taipei Taiwan; ^6^ Department of Kinesiology, Health and Leisure Studies National University of Kaohsiung Kaohsiung Taiwan; ^7^ Department of Bioinformatics and Medical Engineering Asia University Taichung Taiwan

**Keywords:** *GJB2*, *SLC25A13*, variant analysis, whole‐genome sequencing

## Abstract

**Background:**

Cancer is a major cause of death, and its early identification and intervention have potential for clinical actionability and benefits for human health. The studies using whole‐genome sequencing (WGS) and large samples analysis of cancer‐related genes have been rarely done.

**Methods:**

We performed WGS to explore germline mutations in coding and non‐coding areas of cancer‐related genes and non‐coding driver genes and regulatory areas. Structural variants (SVs) was also analyzed. We used several tools and a subgrouping method to analyze the variants in 1491 healthy participants. Moreover, 275 cancer‐related genes sequencing was carried out in 125 cancer patients.

**Results:**

The incidence of familial cancer in the Taiwanese general population is 8.79% (131/1491). Cancer carrier rate of cancer‐related genes is about 7.04% (105/1491) for pathogenic/likely pathogenic variants (P/LP) on ClinVar database only, and 28.24% (421/1491) for P/LP and loss of function variants. The carrier frequencies of cancer‐related genes P/LP on ClinVar database were as follows: 8.40% (11/131), 7.11% (28/394), and 6.83% (66/966) in FC, 1MC, and nMC, respectively. The SVs and non‐coding driver gene variants are uncommon. There are 1.54% (23/1491) of actionable cancer genes in American College of Medical Genetics and Genomics (ACMG), and the germline mutation rate of 275 cancer‐related genes is 7.2% (9/125) in cancer patients including 4.0% (5/125) of actionable cancer genes in ACMG. After analyzing the frequencies of P/LP variants on *GJB2* and *SLC25A13* genes, we suggest that these two genes may not be cancer‐related genes and need be re‐evaluated.

**Conclusions:**

WGS analysis can completely detect germline mutations in cancer carriers. This study use subgrouping approach for samples provides a strategy to study whether a gene or variant is a cancer‐related gene or variant in the future studies.

## Graphical abstract

The strategies for cancer carrier screening are shown. The strategies including participants, targeted genes, and whole‐genome sequencing analyzing approach.

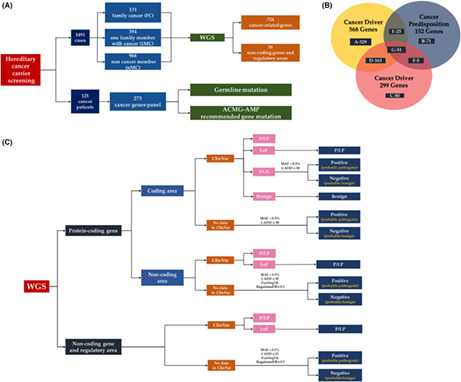

## 1 BACKGROUND

Cancer is a major public health problem worldwide and is the major leading cause of death in the world,^1^, ^2^ and hereditary causes account about 3%–12.6% of adult cancers and 8.5%–10% of childhood cancers.^3^, ^4^, ^5^, ^6^ The effect of hereditary factors on cancer development can be divided into low‐penetrance susceptibility conferred by common germline variants (1.5–2.0‐fold increase in relative risk), moderate penetrance (2.0–5.0‐fold increase in relative risk), and high penetrance predisposition conferred by rare germline variants (>5.0‐fold increase in relative risk).^7^, ^8^, ^9^, ^10^ Moderate and high penetrance predispositions are usually have an autosomal‐dominant inheritance pattern. The inherited genome can be interrogated at any stage of life, enabling prediction of the future risk of cancer.^11^, ^12^, ^13^ Screening for high‐penetrance inherited variants is performed in families with clusters of tumor types, and more than 100 high‐penetrance cancer predisposition genes (CPGs) are known.^7^, ^14^, ^15^, ^16^, ^17^ Many high‐penetrance germline variants increase the risk of a broader range of cancers than classically described.^6^, ^7^, ^11^, ^12^, ^13^, ^14^, ^15^, ^16^, ^17^ High‐penetrance pathogenic (P) variants are found in 5%–10% of unselected patients with cancer, but most inherited predispositions can be attributed to thousands of alleles common in the population that individually provide only a slightly increased risk of cancer.^10^ The best‐characterized cancers now have more than 100 genomic regions associated with risk, accounting for more than 15%–20% of familial relative risk.^10^

Because cancer driver genes (CDGs) play a key role in cancer development,^18^ carriers of germline P/likely pathogenic (LP) variants of these genes will be at risk of cancer. This proposal is supported by the CPGs that are CDGs.^19^, ^20^ CDGs variant‐related familial cancer may be very rare and span many different genes beyond previous studies, and whole‐genome sequencing (WGS) has not been applied to analyze variants of all CDGs, which may explain why germline P variants in many familial cancers have not been identified. More comprehensive methods are needed to detect carriers. In the cancer genome atlas (TCGA) cohort, 8% of adult cancer cases carrying P/LP germline variants in 152 CPGs.^20^ The Pan‐Cancer Analysis of whole Genomes study of the TCGA and International Cancer Genome Consortium identified that 17% of all patients had rare germline variants associated with cancer.^21^ No matter TCGA or ICGC, they were derived from cancer tissues not from the general population. Recently, Rheinbay et al. analyzed driver point mutations and structural variants (SVs) in non‐coding regions across 2658 genomes of different cancers, and their results showed that point mutations and SVs were less frequent in non‐coding genes and regulatory sequences than in protein‐coding genes.^22^ The roles of germline variants of these non‐coding genes and regions in the development of familial cancer or cancer need to be confirmed.

Variants of cancer‐related genes can be detected by whole‐exome sequencing,^20^, ^23^ but this will miss genes with only non‐coding driver variants in cancer‐related non‐coding genes or SVs. WGS can not only identify these non‐coding regions for potential driver events but can also explore changes in non‐coding driver genes.^22^ WGS can also localize SVs, breakpoints, and connections between distinct genomic loci (juxtapositions).

In this study, we collected 152 CPGs,^20^ 299 CDGs,^23^ 568 CDGs,^24^ and the non‐coding driver genes or regions of Rheinbay's study,^22^ totally 724 protein coding genes and 36 non‐coding driver genes or regulatory areas were included. We also analyzed the germline variants of white blood cells (WBCs) in 125 cancer patients using a 275 cancer genes‐panel to explore the germline variant frequency in cancer patients.

## 2 METHODS

### 2.1 Study participants

Thousand four hundred and ninety one participants' data were collected from the Taiwan Biobank (TWB), a general population‐based research database comprising cancer‐free residents aged 30–70 years enrolled at 31 recruitment stations in Taiwan since 2008. Details on the TWB can be found on its official website (https://taiwanview.twbiobank.org.tw/index).^25^, ^26^, ^27^ We subgroup the participants according to the number of cancer cases in the family as FC: more than 2 of 1st degree family members with cancers (131 cases); 1MC: one family member with cancer; nMC (394 cases): no family member with cancer (966 cases). The study was conducted with the approval of the Institutional Review Board (IRB) of China Medical University Hospital (CMUH 108‐REC1‐091), of the IRB on Biomedical Science Research/IRB‐BM Academia Sinica, Taiwan (TWBR10809‐02). All participants were self‐reported as healthy and Han Chinese ancestry, and signed the written informed consent forms.

We retrospectively reviewed the sequence data from 125 cancer patients who underwent genetic testing from January 2017 to July 2021. This study was approved by the IRB of the China Medical University Hospital (CMUH106‐REC1‐047).

### 2.2 DNA extraction

Peripheral blood of enrolled participants was collected into sodium citrate tubes and DNA was isolated using a Chemagic™ Prime™ instrument. DNA length was measured using a Fragment Analyzer (Agilent) and purity was assessed by measuring the optical density (OD) at 260/280 nm. Samples with an OD 260/280 ratio of 1.6–2.0 were considered pure.

### 2.3 A cancer panel including 275 cancer‐related genes for detecting germline mutation in WBCs

We used a commercialized kit (DHS‐3501Z) from QIAGEN Co. (QIAseq Targeted DNA Panel, Human Comprehensive Cancer Panel), which includes 275 cancer‐related genes to analyze the DNAs of WBCs using next‐generation sequencing (NGS) for germline mutations of cases with solid cancers. Totally, there are 125 cases of solid cancers including 40 lung, 30 ovarian, 16 colon, 14 breast, 4 stomach, 3 each with endometrial and urothelial cancers, and other cancer types with less 3. The clinicopathological features of the patients are given in Table S1.

### 2.4 Bioinformatics analysis of variants

We used WGS data released by the Taiwan Biobank (https://taiwanview.twbiobank.org.tw/search).^25^, ^26^ The VCF data were analyzed in our bioinformatics pipeline, which uses the HGVS database to localize the variant in RNA, and protein and official name: (https://annovar.openbioinformatics.org/en/latest/user‐guide/download/) and (http://hgdownload.cse.ucsc.edu/goldenPath/hg19/database/). We used dbSNP to get an official number (https://ftp.ncbi.nlm.nih.gov/snp/archive/b153/VCF/). We used the genomAD (https://gnomad.broadinstitute.org/) and Taiwan biobank (https://www.twbiobank.org.tw/new_web/) databases, and our database, to evaluate the frequencies of variants, and we analyzed variant character using the ClinVar database (https://ftp.ncbi.nlm.nih.gov/pub/clinvar/vcf_GRCh38/),^28^ and the analytic tools CADD (https://ftp.ncbi.nlm.nih.gov/pub/clinvar/vcf_GRCh38/),^29^, ^30^ RegulomeDB (https://www.regulationspotter.org/),^31^ and FunSeq2 (http://funseq2.gersteinlab.org/).^32^ We leveraged Decipher (https://www.deciphergenomics.org/about/downloads/data) and OMIM (https://www.omim.org/downloads) (Licensing and Registration) to explore the relationships of the variants detected with disease. We also used American College of Medical Genetics and Genomics (ACMG) guideline to analyze the reporting genes.^33^

### 2.5 Statistic testing of rare variants of cancer‐related genes for nMC, 1MC, FC, and 688 cancerous tissues

The gene or variant‐based statistic testing of cancer‐related genes in four subgroups were performed for the aberrant frequencies of deleterious variants in nMC, 1MC, FC, and cancer tissues using method as following: We analyzed the variants in the coding regions in cancerous cases and controls considering the 2 × 2 contingency and those variants whose CADD score >25 were further analyzed to study the association of variants in cancer. We calculated the relative risk of variant in cases as compared to control groups to evaluate the level of enrichment in cancer. The risk ratio was defined to be $\mathrm{RR}=\frac{a/\left(a+b\right)}{c/\left(c+d\right)}$ and adjusted chi‐square test was used to identify significant associations. The R‐package of “epitools” was used to calculate RR of each variant in our study.

### 2.6 Confirmation of variants by PCR and direct sequencing

Primers for direct sequencing validation were designed using Primer3 software. The PCR primers used are shown in Table S2. PCR amplifications were performed using ProTag Plus DNA Polymerase (Protech Technology Enterprise) following the manufacturer's instructions and our previous study.^34^

## 3 RESULTS

**FIGURE 1:**
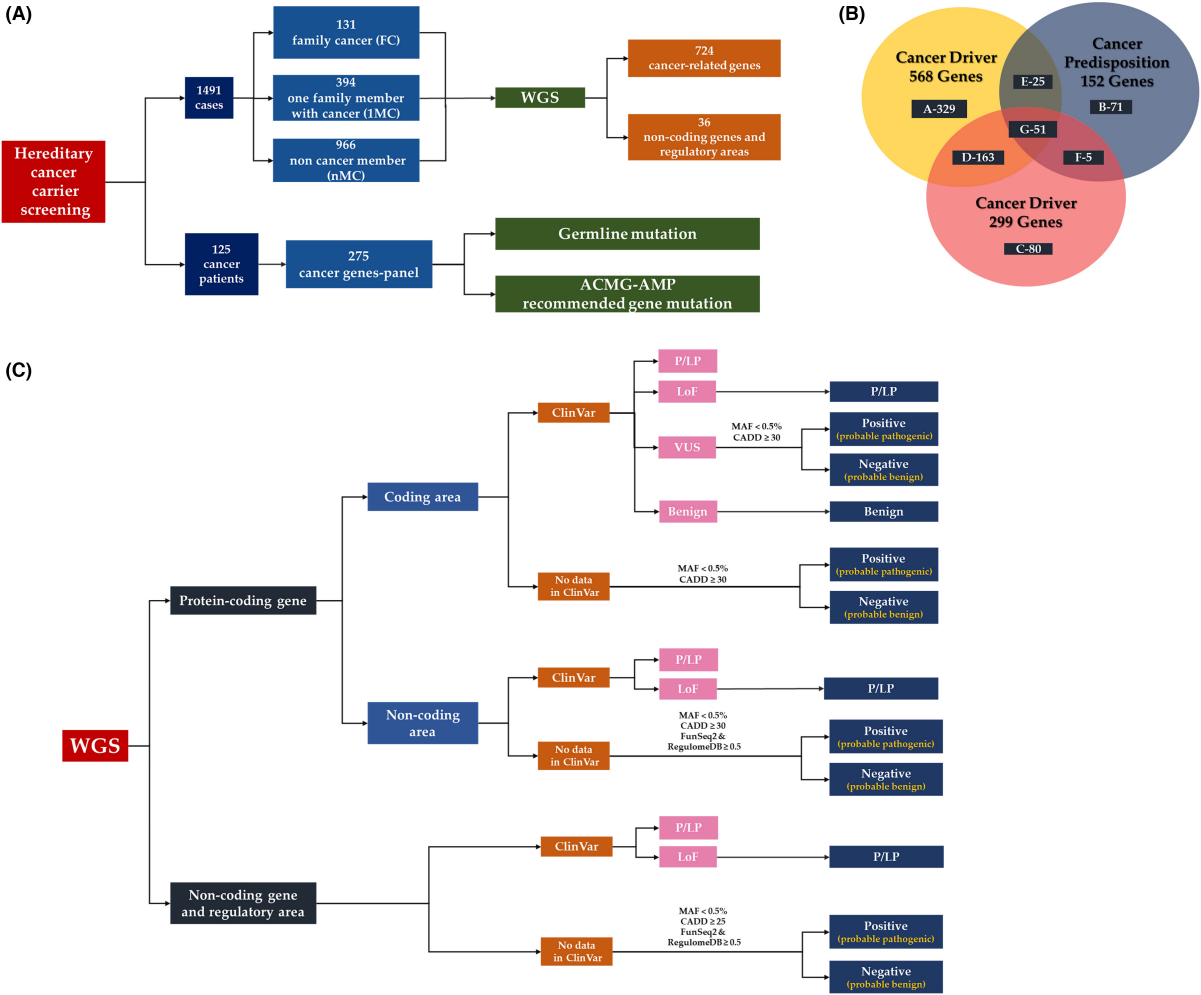
The strategies and analyzing methods of this study are shown. (A) The strategy, participants, targeted genes, and analyzing approaches are shown. (B) Relationships among 152 cancer predisposition genes, 299 cancer driver genes, and 568 cancer driver genes are shown. (C) WGS analysis methods. We analyzed variants of coding and non‐coding areas in protein‐coding genes, and non‐coding genes and regulatory areas using the ClinVar database, and prediction tools (e.g., CADD, RegulomeDB, and Funseq2) to analyze null variants. WGS, whole‐genome sequencing.

The strategy, participants, targeted genes, and analyzing approaches of this study are shown in the Figure 1. Totally, 1491 healthy participants and 125 cancer patients were included, and the healthy participants were further subclassified into FC, 1MC, and nMC according to the cancer occurrence in the family member, and then they were subjected for 724 cancer‐related genes, and 36 non‐coding driver genes and regulatory areas (Figure 1A). The relationships among 152 CPGs, 299 CDGs, and 568 CDGs are shown in Figure 1B. The detailed relationships of these panel genes are shown in Table S3. The strategies of WGS analysis are shown in Figure 1C.

We analyzed variants of coding areas and non‐coding areas including introns, promoters, and regulatory elements using the ClinVar, TCGA, and COSMIC databases for known variants, and we used three prediction tools, CADD for coding areas, and CADD, RegulomeDB, and FunSeq2 for non‐coding areas, to analyze null variants on ClinVar database. For SVs, we used ClinVar and Decipher databases to explore the clinical significance.

We select the targeted variants using ClinVar P/LP, or CADD ≥30 and MAF <0.5% for coding areas, and CADD ≥30 and MAF <0.5% and FunSeq2 ≥0.5 and RegulomeDB ≥0.5 for non‐coding areas as a selected criteria.

### 3.1 Stratification of participants

We collected 1491 WGS data from the general population of Taiwan Biobank. The male to female ratio was near 1 (744/747), and the age range was 30–70 years, and the detail of demographic data was shown in Table S4.

### 3.2 Variant detection of coding areas and non‐coding areas in 724 cancer‐related genes

Totally, 724 cancer‐related genes (152 CPGs + 299 CDGs + 568 CDGs and excluding the overlap genes) were collected in this study, and the summarized results which include P/LP variants in the ClinVar database, or CADD score ≥30 are shown in the Table 1A. The detailed variants are shown in Tables [Link], [Link].

**TABLE 1:** Distribution of case numbers of important variants of 724 cancer‐related genes in (A) 1491 participants and (B) three subgroups

(A)							
		Coding area			Non‐coding area		
		ClinVar	None		ClinVar	None	
		P/LP	CADD ≧30	LoF	P/LP	CADD ≧30	Splicing
WGS	724 cancer‐related genes	74	573	177	31	300	139

(B)																		
Subgroup (cases) Targeted panel	FC (131 cases)						1MC (394 cases)						nMC (966 cases)					
Variant types	Coding area			Non‐coding area			Coding area			Non‐coding area			Coding area			Non‐coding area		
	ClinVar	None		ClinVar	None		ClinVar	None		ClinVar	None		ClinVar	None		ClinVar	None	
	P/LP	CADD ≧30	LoF	P/LP	CADD ≧30	Splicing	P/LP	CADD ≧30	LoF	P/LP	CADD ≧30	Splicing	P/LP	CADD ≧30	LoF	P/LP	CADD ≧30	Splicing
724 cancer‐related genes	9	67	17	2	30	9	19	154	46	9	81	41	46	352	114	20	189	89

*Note*: The important variants were selected based on Figure 1C approach.

Abbreviation: P/LP, pathogenic/likely pathogenic.

In the coding areas of 724 cancer‐related genes, 74 participants have ClinVar P/LP (Table S5), and 573 participants have CADD score ≥30 variants with no ClinVar data which we consider as possible pathogenic (PP) variants (Table S6). Among the 573 participants, 177 have loss of function variants.

In the non‐coding areas of 724 cancer‐related genes, there are 45 participants who have ClinVar P/LP (Table S7), and 300 participants have CADD score ≥30 variants with no ClinVar data which we consider as PP variants (Table S8). Among the 300 participants, 139 participants have CADD ≥30 variants resulting from disrupting splicing machinery.

From these results, we suggest the cancer carrier frequency of driver genes of Taiwanese general population is 7.04% (105/1491) for ClinVar P/LP, or 28.24% (421/1491) for all the important variants including P/LP, stop gain or loss, frameshift, and splicing‐disrupted (Table 1A). In these frequencies, we do not include the SVs and non‐coding genes driver variants that may result in slightly underestimating the frequencies.

The variants of P/LP of ClinVar in 724 cancer‐related genes in FC, 1MC, and nMC are shown in Table 1B. The frequencies of several P/LP variants of *GJB2* gene were higher in 1MC and nMC than FC (p.Leu79X variant, 0.76%, 1.27%, and 1.35% for FC, 1MC, and nMC, respectively; for c.761G>GCGTT and c.549CAT>C frameshift variants were found only in 1MC and nMC) and the frequency of the P variant Val37Ile was very high among FC, 1MC, and nMC (>16% in all groups) (Table S5). For *SLC25A13*, the frequency of c.1043TCATA>T was 1.53%, 1.52%, and 1.55% for FC, 1MC, and nMC, respectively, and there was no difference in frequency among FC, 1MC, and nMC. We used statistic test for these two genes, and the results showed there are no significant different between FC, nMC or 1MC. Similar results were found, after we further used 688 different cancer tissues to confirm the results (Table 2; Table S9). Therefore, these two genes are not likely cancer‐related genes.

**TABLE 2:** The statistics of the variants with ClinVar P/LP or CADD ≥25 between cancer and other non‐cancer groups

Gene name	dbSNP	Cancer versus nMC (*p*‐value)	Cancer versus nMC (adjusted *p*‐value)	Cancer versus 1MC (*p*‐value)	Cancer versus 1MC (adjusted *p*‐value)	Cancer versus FC (*p*‐value)	Cancer versus FC (adjusted *p*‐value)
*GJB2*	rs776335807	NA	NA	0.1953612	0.716	NA	NA
*GJB2*	rs111033204	0.7168588	0.765	0.8846106	0.885	0.5281364	0.658
*GJB2*	rs80338943	0.8147019	0.815	0.814702	0.885	0.65822	0.658
*GJB2*	rs587783644	0.4083326	0.503	NA	NA	NA	NA
*SLC25A13*	rs80338725	0.006740853	0.108	0.08331204	0.716	0.3176162	0.658
*SLC25A13*	rs752235032	0.4083326	0.503	NA	NA	NA	NA
*SLC25A13*	rs139149160	0.08690023	0.503	0.2740806	0.754	0.5281364	0.658
*SLC25A13*	rs80338722	0.4083326	0.503	0.1953612	0.716	NA	NA
*SLC25A13*	rs776461118	0.4083326	0.503	NA	NA	NA	NA
*SLC25A13*	rs949468946	0.2262096	0.503	0.4395195	0.802	0.655743	0.658
*SLC25A13*	rs398122839	0.2262096	0.503	0.7119804	0.885	0.2027535	0.658
*SLC25A13*	rs1484296612	0.4083326	0.503	NA	NA	NA	NA
*SLC25A13*	rs80338720	0.3988902	0.503	0.5106532	0.802	0.6458937	0.658
*SLC25A13*	rs751343245	0.2421313	0.503	NA	NA	0.02470359	0.247
*SLC25A13*	rs879255503	0.4083326	0.503	NA	NA	NA	NA
*SLC25A13*	rs80338717	0.6843908	0.765	0.7706653	0.885	0.2053228	0.658
*SLC25A13*	rs1254503252	0.2262096	0.503	0.4395195	0.802	0.655743	0.658

Abbreviation: P/LP, pathogenic/likely pathogenic.

A ClinVar P variant in the *SBDS* gene had a >0.2% allelic frequency in gnomAD and our three groups. Therefore, this variant is not a moderate‐ or high‐risk variant, and not included in the analysis of cancer‐related gene carrier frequency (Table S7). After removing these variants, the cancer‐related gene carrier frequencies of P/LP on ClinVar database were 8.40% (11/131), 7.11% (28/394), and 6.83% (66/966) in FC, 1MC, and nMC, respectively (Table 1B).

Several ClinVar P/LP variants were found in the non‐familial cancer groups, such as *ATM* c.1787CAA>C, p.K468X, and *RAD51D*, c.531T>TTA, p.K111IX, in 1MC and nMC; *BRCA2* c.8242C>T, p.Ser2670Leu, and *MSH6* c.3378C>T, p.Arg1076Cys, in 1MC; *DNMT3A* c.2982G>A, p.Arg882Cys, *BLM* c.1653G>GA, p.G512GX, *FANCA* c.1018TGTGA>T, p.TH329X, *FANCI* c.1926C>T, p.Arg614*, *RNF213* c.14572G>A, p.Arg4810Lys, and *WRN* c.748TAA>T, p.K167X, in the nMC (Table S5). Therefore, we suggest that cancer carrier screening should not be limited to FC, and must include other non‐familial cancer groups. Additional, several P/LP variants were found only in the FC group, such as *JAK2* c.2343G>T, p.Val617Phe, and *LZTR1* c.379C>CG, p.T7TX (Table S5), which may be causal variants.

We also evaluated the carrier frequency of reporting of secondary findings in ACMG, and the results showed that the frequency of P/LP of ClinVar in 1491 participants is 1.54% (23/1491) for 28 reporting cancer genes in ACMG‐AMP guideline (Table S10).^33^

### 3.3 Analysis of non‐coding genes and regulatory areas

In the non‐coding driver genes and regulatory areas of Rheinbay's study, there are 13 participants having variants CADD score ≥25 (Table S11). The results were shown that 13 variants were found including three at *Hes1‐1*, five at *HIST1H2AM*, and one at *SDCCAG8‐3* promoters, respectively, and four at lncRNA *RMRP*. The promoter variant of *SDCCAG8‐3* has a high CADD score, but has conflicting interpretation on ClinVar database. These variants may be not cancer‐related.

### 3.4 SV analysis in cancer‐related genes

We found 2002 SV events in the 724 cancer‐related genes from 1488 NGS samples and selected rare SVs (those in ≤2 samples), that involve 603 oncogenes (209 deletions, 43 duplications, 339 insertions, and 12 inversions) and 876 tumor suppressor genes (284 deletions, 66 duplications, 508 insertions, and 18 inversions) (Table S12). Summary and details of rare SVs in subgroups are shown on Table S13, and most of them are located on the intronic regions. We specifically focused on the six SVs that involved exon areas of targeted genes, including three oncogenes, two tumor suppressor genes, and one biphase gene (Table S14). These function disrupted exon‐related SVs may play a role as cancer driver, which need to be further studied.

### 3.5 Results of a special cancer family

**FIGURE 2:**
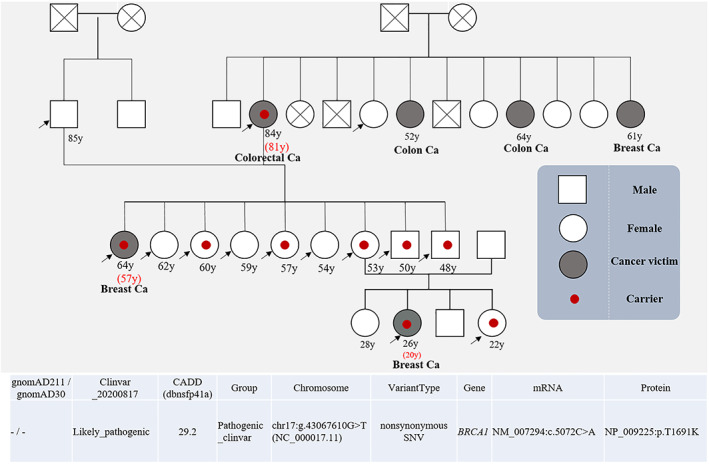
Pedigree and genetic analysis results of a cancer family. The arrow indicates the members who took WGS analysis. Red numbers are the times of cancer detection. Red dot is carrier cases under WGS analysis. WGS, whole‐genome sequencing.

We asked a participant with six family members with cancer spanning three generations for consent to analyze his family. We performed WGS of 14 members of this family and found nine with the c.5072C>A, p.T1691K mutation on the *BRCA1* gene (Figure 2). This family had two breast cancer members including one at a young age (20 years old), with triple negative breast cancer, one with colorectal cancer, and three females >50 years old with no cancer. From these results, we suggest that other factors may play a modification role in the time of cancer development.

### 3.6 Evaluation of germline mutation of cancer patients

We explored the germline mutations of patients with solid cancer using a cancer panel with 275 cancer‐related genes, and this panel contains 22/28 of reporting of secondary findings in ACMG‐AMP.^33^ The results showed that 7.2% (9 of 125) cancer patients have mutations on 275 cancer‐related genes, and 4.0% (5 of 125) have reporting of secondary findings in ACMG‐AMP (Table S15).

### 3.7 Confirmation of base and SVs by PCR and direct sequencing

**FIGURE 3:**
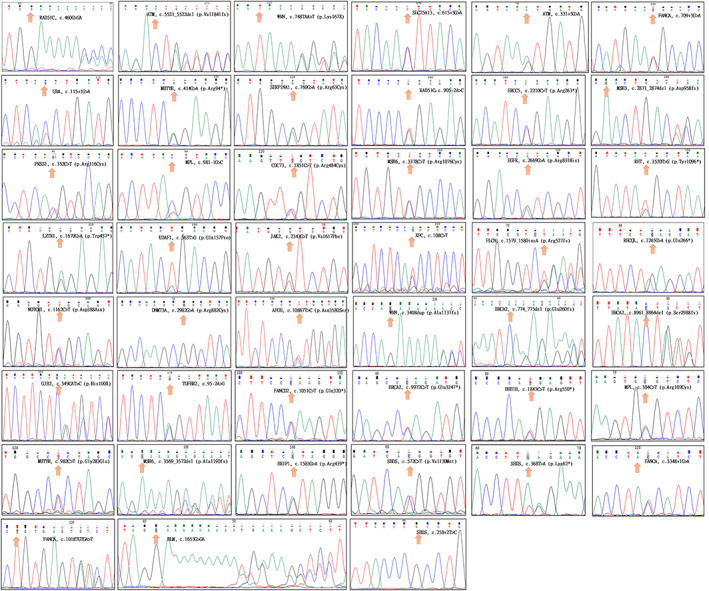
Sanger sequencing of pathogenic or likely pathogenic variants.

**FIGURE 4:**
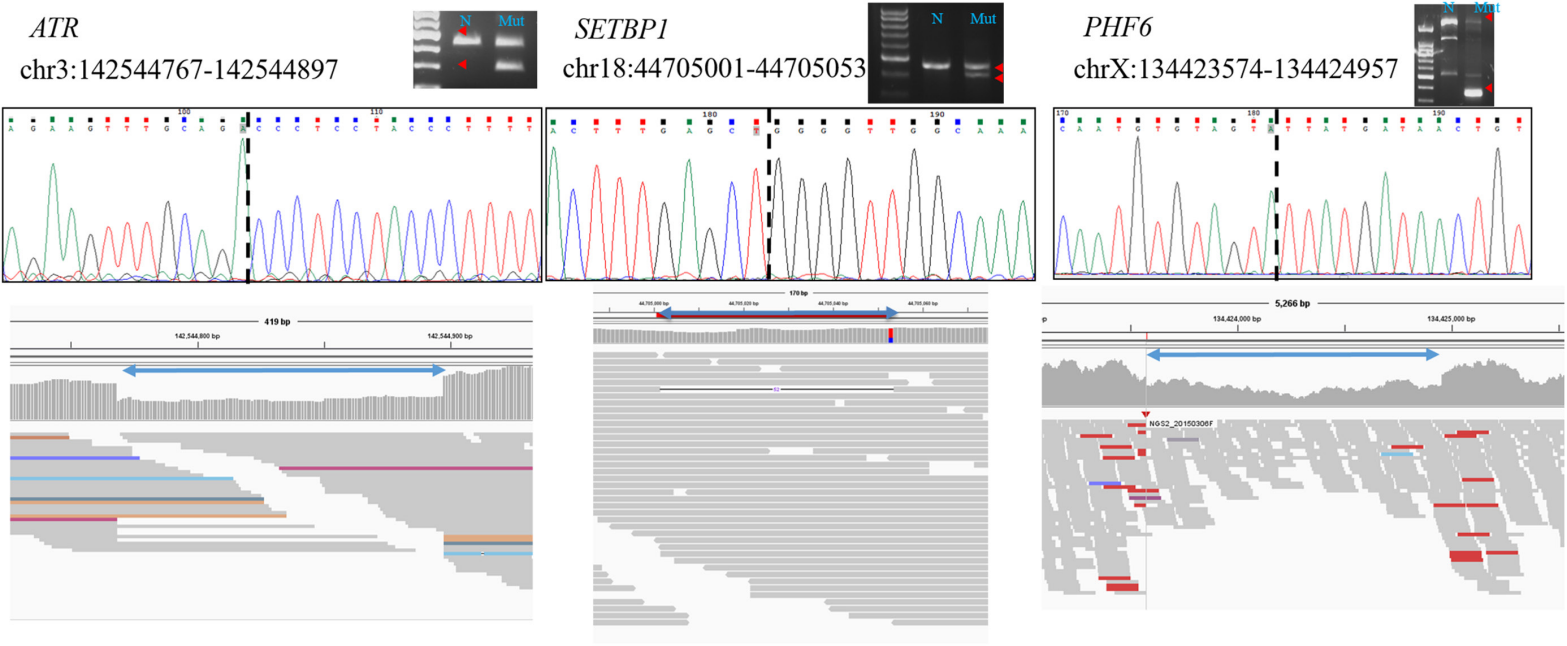
Sanger sequencing of structural variants.

We sequenced >20% of the variants to confirm the NGS results (Figure 3), and we also confirmed the SVs using Sanger sequencing (Figure 4). The results showed that the false positive rates were about 5% for single base variants and 25% for SVs. From these results, we suggest that the confirmation of driver variants in needed.

## 4 DISCUSSION

CDGis the major determinant of cancer, and the number differs according to the approach used. In total, we analyzed 724 cancer‐related genes, and 36 non‐coding driver genes and regulatory areas including the studies of Rahman et al.,^19^ Bailey et al.,^23^ Rheinbay et al.,^22^ and Martinez‐Jimenez et al.^24^ to detect carriers of cancer‐related gene mutations using GS, and our results showed that this approach is a more comprehensive panel and may not lose reporting cancer genes in ACMG, and our approach is a WGS‐based method which can cover all the cancer‐related genes for the new changes of ACMG‐AMP in the future.^33^

The frequencies of several detrimental variants of cancer‐related genes (such as *GJB2* and *SLC25A13*) were higher in the non‐familial cancer group than the familial cancer group. We used the data of subgrouping, and found no differences between FC and non‐FC, and suggest these genes or variants may not play a role in the cancer development or not a true cancer‐related genes. We further used statistic test and cancer tissues to confirm the subgrouping findings. Therefore, we suggest that *GJB2* and *SLC25A13* may not be cancer‐related genes according to the results of statistic test. We suggest this subgrouping approach could be used to screening whether a gene or variant is a cancer‐related gene or variant, and then using statistic test to confirm the finding. The power of screening will be increased after collected more data in the subgroups.

There are many genetic testing including different number of genes to explore the candidate gene in different cancer families, and the positive results are usually not high, which may result from ethnic, population difference and gene numbers including type of genes.^35^ In this study, in the non‐familial cancer groups, there are over 6% having P variants of cancer‐related genes, most of them are the causes of familial cancer and high penetrance. Therefore, WGS should be used for screening for hereditary cancer to avoid false negative finding or de novo mutation. In addition, the number of known hereditary cancer genes has increased and it has been demonstrated that germline susceptibility to cancer is more prevalent than formerly believed; our findings also confirm these concept.^36^, ^37^

We used prediction tool CADD as a major tool to analyze the null variants on ClinVar database, this tool is a popularly used evaluator of variant deleteriousness that can effectively and efficiently cataloged causal variants in genetic analyses, especially for highly penetrants of severe Mendelian disorders. CADD has integrated more than 60 genomic features to annotate the variants, and can score human single nucleotide variants and short insertion and deletions anywhere in the reference assembly.^29^, ^30^ We used CADD score ≥30 and MAF <0.5% as a variant selection standard for null variants in ClinVar database of coding areas of protein‐coding genes, or CADD ≥30 and MAF <0.5% and FunSeq2 ≥0.5 and RegulomeDB ≥0.5 as a variant selection standard for null variants in ClinVar database of non‐coding areas of protein‐coding genes, or CADD score ≥25 for non‐coding driver genes and regulatory areas as an important P variants for cancer‐related genes. Using these strict criteria, we were able to avoid over‐presented cancer related variants, but we may lose some false negative variants. There are over 40 different in silico programs for variant prediction, CADD is only one of them. In addition to CADD, we also used RegulomeDB, and FunSeq2 to improve the prediction accuracy.

Driver‐gene screening may include many low‐ to moderate‐penetrance genes, but the optimal strategy for managing carriers of low to moderate‐penetrance mutations is unclear. Many variants of unknown significance will be detected using WGS. More data on cases with driver gene mutations is needed to evaluate their clinical significance and prevent overdiagnosis.^38^, ^39^

WGS enables detection of P and LP variants in cancer‐related genes to identify cancer carriers, and WGS is rapid and more cost‐effective than other methods used for cancer carrier screening, such as WES.^40^

## AUTHOR CONTRIBUTIONS

Conceptualization: Ya‐Sian Chang, Jan‐Gowth Chang; Data curation: Hou‐Wei Chu, Shun‐Jen Chang, Chieh‐Min Chang; Formal analysis: Dy‐San Chao, Yu‐Pao Chou, Hon‐Da Chen, Ya‐Sian Chang, Jan‐Gowth Chang; Funding acquisition: Jan‐Gowth Chang; Methodology: Dy‐San Chao, Chin‐Chun Chung, Yu‐Pao Chou, Chia‐Li Lin, Hon‐Da Chen,Ting‐Yuan Liu; Project administration: Center for Precision Medicine, CMUH; Resources; CMUH, and TWB; Software: CADD, REVEL, RegulomeDB, and FunSeq2; Supervision: Jan‐Gowth Chang; Validation: Ya‐Sian Chang, Chia‐Li Lin; Visualization: Yu‐Hsuan Juan; Writing—original draft: Ya‐Sian Chang, Jan‐Gowth Chang; Writing—review & editing: Ya‐Sian Chang, Jan‐Gowth Chang

## CONFLICT OF INTEREST

None.

## ETHICAL APPROVAL STATEMENT

The study was conducted with the approval of the Institutional Review Board of China Medical University Hospital (CMUH 108‐REC1‐091), of the IRB on Biomedical Science Research/IRB‐BM Academia Sinica, Taiwan (TWBR‐02). The written informed consent has been obtained from all the patients included in this study.

## Supporting information


Table S1

Table S2

Table S3

Table S4

Table S5

Table S6

Table S7

Table S8

Table S9

Table S10

Table S11

Table S12

Table S13

Table S14

Table S15

## Data Availability

Raw data were generated from Taiwan Biobank (https://www.twbiobank.org.tw/new_web_en/about‐export.php). The datasets generated during and/or analyzed during the current study are not publicly available due to the data containing sensitive information about patients but are available from the corresponding author upon reasonable request.
